# Feasibility of Privacy-preserving Locally Hosted Language Models for Assessing Medical Student Reflective Writing

**DOI:** 10.1007/s40670-026-02865-7

**Published:** 2026-09-01

**Authors:** Rick Rejeleene, Jessica N. Byram, Richard M. Frankel, J. Harry Isaacson, Neil B. Mehta

**Affiliations:** 1 Cleveland Clinic Lerner College of Medicine at Case Western Reserve University, Cleveland, OH USA; 2 Department of Medical Education, Tufts University School of Medicine, 136 Harrison Ave., Boston, MA USA; 3 Department of Medicine, Indiana University School of Medicine, Indianapolis, IN USA

**Keywords:** LLM, Reflective writing, Feedback

## Abstract

Reflective writing assessment in medical education is labor-intensive and raises data privacy concerns that preclude cloud-based AI solutions. We conducted a feasibility study using a locally-hosted, open-weight large language model (LLM) deployed on institutional servers to assess medical students’ reflective writing. The model evaluated 10 de-identified student narratives using two published rubrics across three independent passes, generating rubric-aligned scores and formative feedback. Scores were consistent across passes and rubrics with no statistically significant differences. Expert faculty rated LLM-generated feedback as meeting most quality criteria (mean 5.48/7). These findings support feasibility of a privacy-preserving, locally-hosted LLM for scalable reflective writing assessment.

**Supplementary Information:**

The online version contains supplementary material available at https://doi.org/10.1007/s40670-026-02865-7.

## Background

Reflective writing supports self-regulation, professional identity formation, and compassionate patient care, and is increasingly embedded in medical school curricula [1–2]. To maximize the benefit of reflective writing on learning and practice, feedback is necessary to help students engage in the writing [3] and to develop skills in reflective practice [4]. However, an ongoing challenge is that evaluating reflective writing as a component of reflective practice is labor-intensive, subject to inter-rater variability, and often produces feedback that is inconsistent or superficial [5–7].

While Natural Language Processors (NLPs) have been used to extract insights from textual data and automate analysis of reflections, they are limited in their ability to provide narrative evaluations [8–10]. Large Language Models (LLMs) offer a potential solution for standardizing narrative assessment at scale both quantitatively and qualitatively [11–15]. Institutional concerns regarding data privacy and the protection of sensitive student narratives frequently may preclude the use of cloud-based models like GPT-4. To address these challenges, we conducted a feasibility study using a “local-first,” on-premises LLM to evaluate the intra-model reliability and formative feedback quality of a secure model when assessing medical student reflective practice essays.

## Activity

### Study Design

**Fig. 1:**
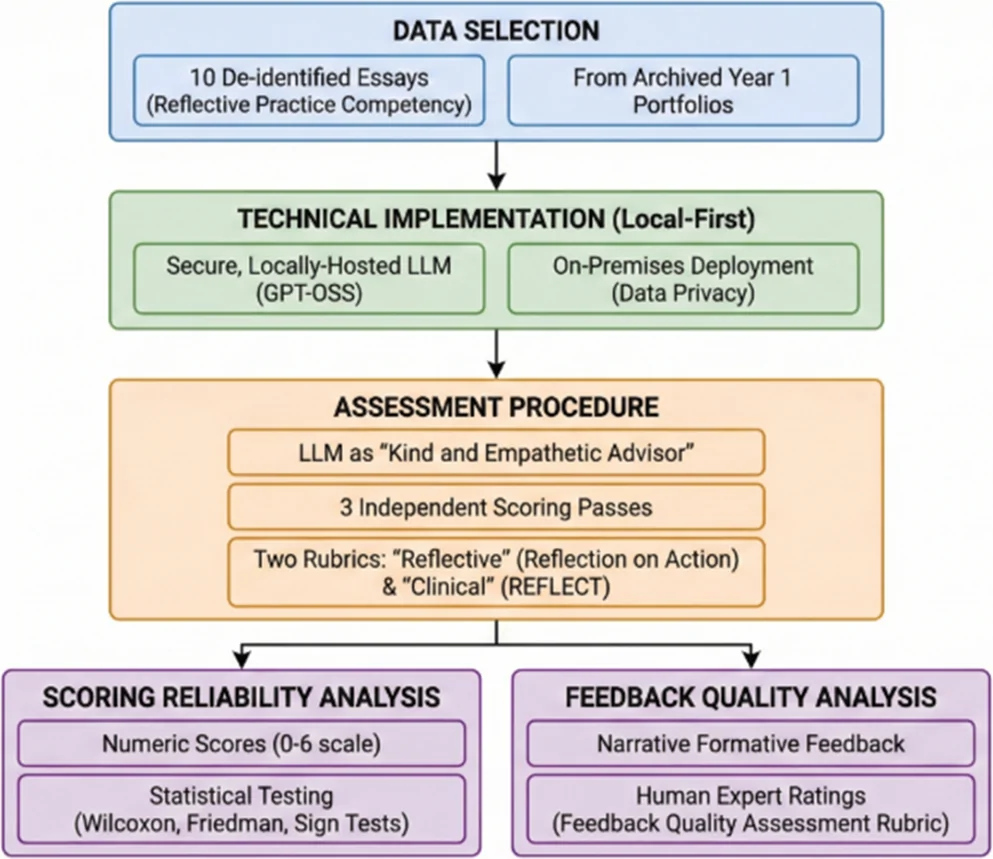
Overview of the pilot study procedure for evaluating LLM-generated assessment of reflective practice essays

The competency-based curriculum at Cleveland Clinic Lerner College of Medicine (CCLCM) requires students to submit summative reflective portfolio essays in years 1, 2 and 4, citing narrative evidence that they have met the milestones for each of the school’s nine competencies, including Reflective Practice. We randomly selected 10 de-identified Reflective Practice essays, stratified by gender, from archived Year 1 portfolios (Fig. 1). As summative portfolio essays, these narratives are milestone-anchored and structurally constrained by length requirements, representing students’ most considered reflective work, a population of essays likely more uniform in quality and depth than reflections produced under lower-stakes conditions.

The study was approved by the Cleveland Clinic/Case Western University institutional review board.

## Technical Implementation

To address privacy and scalability, we developed a secure Django-based local-first web application for assessing medical student reflective writing using an open-weight language model, GPT-OSS-20B. The application and local model inference were hosted on an institutionally managed Apple MacBook Pro 16-inch, November 2023 model, equipped with an Apple M3 Max system-on-chip, 16-core CPU, 40-core GPU, 16-core Neural Engine, and 64 GB unified memory, running macOS Sequoia 15.7.5. This local institutional deployment allowed de-identified student narratives to be processed without transmission to external cloud-based LLM services.

The system used a reproducible workflow involving secure essay upload, rubric-based prompting, automated score generation, formative narrative feedback generation, and storage of outputs for longitudinal review. This architecture allowed the institution to test privacy-preserving LLM-assisted reflective writing assessment while maintaining local control over sensitive educational data.

## Data and Experimental Procedure

The local LLM was configured using the following “system prompt” defining its persona to focus on a feed forward approach to narrative feedback [15]: “You are a kind and empathetic advisor or mentor and coach for a medical student. You are helping them strengthen their reflective ability by providing formative feedback on essays they have written about their reflective practice(s). Use the reflective ability rubric as a guide and write narrative formative feedback to the student summarizing their strengths in reflection and ask them questions to nudge them to get to a deeper level of reflection.” It was tasked with generating numeric scores and formative narrative feedback based on two established assessment tools: the Reflection on Action rubric [16] and the REFLECT rubric [2]. These rubrics were designated as the “Reflective” and “Clinical” rubrics, respectively. The essays were evaluated using both rubrics across three independent passes. Output/feedback was scored quantitatively on a 6-point scale (0 = no response or reflection to 6 = exceptional or critical reflection) with 0.5 increments.

## Evaluation of Reliability and Feedback Quality

Evaluation focused on two dimensions: reliability and quality. To assess reliability, the model scored the same set of essays and provided narrative feedback across three independent passes to test for consistency and rubric-agnosticism. To assess quality, two experienced human faculty who were blinded rated the LLM-generated narrative feedback. They utilized a “Feedback Quality Assessment Rubric.”

To find significant differences among the rubric scores, we used three non-parametric statistical tests executed in R version 4.5.2 (R Core Team, 2025). Wilcoxon signed-rank test was used to evaluate paired differences between the clinical and reflective rubric for each essay. Friedman tests evaluated differences in reflection scores across the three passes. Sign tests analyzed differences across paired rubric scores.

## Development of Feedback Quality Assessment Rubric

To evaluate the quality of the LLM narrative feedback, a quality assessment rubric was developed as none of the existing frameworks or rubrics entirely met our needs [16–18]. Existing tools either address the quality of faculty-written performance narratives in workplace-based assessment contexts [16, 18] or provide frameworks for guiding human raters rather than evaluating AI-generated output [15]. None were designed to assess formative feedback produced by a language model in response to student reflective writing while the one pilot study [13] that examined ChatGPT feedback quality did so without attention to reflective writing or rubric alignment.

The Feedback Quality Assessment Rubric includes seven criteria related to performance, evaluation of reflective ability and lessons learned, and highlighting areas for improvement (see Appendix for a worked example with LLM narrative feedback). The LLM-generated narrative feedback (*n* = 20) was independently scored by two evaluators with extensive experience providing narrative feedback. Interrater reliability was evaluated using Cohen’s Kappa and descriptive statistics calculated prevalence of rubric criteria in the LLM feedback (SPSS v.31; IBM).

## Results

### Scoring Consistency and Reliability

Scores ranged from 4.0 to 5.0 on a 0–6 scale, with occasional 0.5-point fluctuations (Table 1). No significant differences were observed between rubrics across passes (*p* = 1.00). Friedman testing showed no differences across repeated passes (χ² = 3.47, *p* = 0.6), and the Sign test confirmed no directional scoring bias (*p* = 1.00). The Sign test p-value of 1.00 reflects the complete absence of directional scoring differences across rubrics, consistent with the narrow score range observed.

**Table 1:** GPT-OSS scores of student reflections across three passes and the Clinical and Reflective Rubrics

Essay	Pass 1 – Clinical	Pass 1 – Reflective	Pass 2 – Clinical	Pass 2 – Reflective	Pass 3 – Clinical	Pass 3 – Reflective
1	5.0	4.5	4.5	4.5	4.5	5.0
2	4.5	4.5	4.5	4.5	4.5	4.5
3	4.0	4.5	4.5	4.5	4.0	4.0
4	4.5	4.5	5.0	5.0	4.5	4.5
5	4.5	4.5	5.0	5.0	4.5	4.5
6	4.0	4.5	4.5	4.5	4.5	4.5
7	4.5	5.0	4.5	4.5	5.0	4.5
8	4.5	4.5	4.5	4.5	4.5	4.5
9	4.5	4.0	4.5	4.0	4.5	4.5
10	4.5	4.0	4.5	4.5	4.5	4.5

Reflective Ability Scores on a 0–6 scale with 0.5-point increments (0 = no response or reflection; 1 = minimal or superficial; 2 = basic description with minimal analysis; 3 = thoughtful action but limited reflection; 4 = reflection; 5 = advanced reflection considering multiple perspectives; and 6 = exceptional or critical reflection)

## Feedback Quality Assessment

LLM feedback met a mean of 5.48 of seven quality criteria. Criterion 6 (prompts for further reflection) was present in all responses; other criteria ranged from 55 to 88% prevalence (Table 2). Inter-rater agreement was fair (κ = 0.21), which may reflect rubric ambiguity or the absence of formal rater calibration prior to scoring, as well as the inherent subjectivity of evaluating narrative reflections.

**Table 2:** Prevalence of Criteria in the Feedback Quality Assessment Rubric across LLM feedback (*n* = 40)

Feedback Criteria	Mean	SD
1. Feedback references specific themes and/or behaviors from the reflection.	0.85	0.362
2. Feedback comments on reflective ability (i.e., what was done and/or what was done well).	0.65	0.483
3. Feedback makes specific reference to emotions and/or feelings described in the reflection.	0.80	0.405
4. Feedback comments on the writer’s description of the lessons learned.	0.55	0.504
5. Feedback draws attention to parallel processes (e.g., situates the reflection in a related personal, professional, religious, and/or societal contexts).	0.75	0.439
6. Feedback provokes consideration of change(s) in the writer with questions/prompts for further consideration.	1	0
7. Feedback highlights areas for improvement in their reflective practice (e.g., deepen ability to see, understand and/or act on one’s own perspectives).	0.88	0.335

## Discussion

These findings demonstrate the feasibility of using a locally hosted, privacy-preserving open-weight LLM to automate reflective writing assessment (scoring and feedback). Using a “local-first” infrastructure, we successfully addressed the tension between automated scaling and data privacy, ensuring sensitive narratives remain within the institution’s secure environment. This enables rubric-aligned feedback to promote reflective practice and reduce workload for evaluators. The statistical stability observed suggests high intra-rater reliability, however, we observed a distinct “central tendency” in the quantitative scoring, where scores clustered tightly between 4.0 and 5.0. This may be an artifact of the LLM’s tendency to remain agreeable, or the homogeneous performance of these learners. The latter is plausible as students have completed three formative reflections prior to submitting the summative essays used in this study. Distinguishing between algorithmic bias and true cohort homogeneity will require further testing on a broader range of essays.

Our preliminary evaluation, which demonstrated high intra-model reliability and high-quality feedback ratings from expert faculty, informs several critical avenues for future investigation.

Validation of Numeric Scoring. While our pilot confirmed that the LLM’s narrative feedback was rated highly by faculty experts, we need to establish the validity of the *numeric* assessment. A concordance study comparing the LLM-generated scores against trained faculty raters is planned to ensure that the model’s grading severity and interpretation of the rubrics align with pedagogical intent and human consensus.

Student Perception and Impact. Studies suggest that some students cannot differentiate AI versus human feedback [10]. When prompted to compare narrative feedback from longitudinal facilitators or ChatGPT-4, however, they rated the LLM-generated feedback significantly higher on its ability to help identify strengths/areas of improvement and provide actional steps for improvement [13]. Our next step will be to pilot the system with a live cohort of students to determine if the local AI-generated feedback produces actionable results that promote desired changes in reflective ability.

Model Optimization and Scaling. We utilized GPT-OSS for this study to ensure data containment; future work will benchmark this model against other open-source, weight-accessible models (e.g., Llama 3 or Mistral) to determine the optimal balance between inference speed and reasoning capability using an expanded dataset.

Human-in-the-Loop Integration. We view this tool as an augmentation for faculty, not a replacement. Similar to Hanlon and colleagues who used an NLP to identify reflective behavior in narratives to cue feedback from faculty [8], we will pilot a “human-in-the-loop” workflow where the LLM provides a preliminary score and draft feedback for faculty to review and assess any reductions in administrative burden and quality of grading from this workflow.

Limitations. Local LLMs may not have the same performance characteristics as web-based LLMs like ChatGPT; our pilot was small, testing 10 essays, focused on using a single LLM (GPT-OSS), and we only examined intra-model consistency, rubric-agnostic behavior. The “fair” inter-rater agreement (κ = 0.21) among human faculty on feedback quality likely reflects the inherent subjectivity of evaluating narrative reflection. Larger, multi-model studies are required to establish broader reliability and to refine the clarity of the quality assessment criteria.

In this feasibility study, a locally hosted LLM produced stable, rubric-aligned evaluation of reflective writings across two established rubrics on three independent scoring passes. Absence of statistically significant differences between rubrics or across passes suggests reliable intra-model consistency and no scoring bias. This supports feasibility of on-premises locally run LLMs to generate reflective scores and formative feedback, while preserving privacy of the learner and reducing the time burden on faculty. A local LLM offers a scalable blueprint for other medical schools facing similar assessment challenges.

## Supplementary Information

Below is the link to the electronic supplementary material.

Supplementary Material 1.

## Data Availability

The datasets generated during and/or analyzed during the current study are available from the corresponding author on reasonable request.
