# Supplementary material for: Feasibility of Privacy-preserving Locally Hosted Language Models for Assessing Medical Student Reflective Writing
Source: Med Sci Educ. 2026 Sep 1;36(5):2157–61. doi: 10.1007/s40670-026-02865-7 (PMC13723480; doi:10.1007/s40670-026-02865-7)
Supplement: Supplementary file 1 [file 40670_2026_2865_MOESM1_ESM.docx]

**APPENDIX**

**Framework for Developing the Feedback Quality Assessment Rubric**

Our rubric items were based on the BEGAN framework [1], quality indicators developed by Chakroun and colleagues [2], and the EFeCT Feedback Scoring Tool [3]. The BEGAN framework includes a five-step process with prompts and suggestions to assist in crafting feedback to students on their reflective writing. Steps include gut reactions, initial impressions, identifying key themes, emotions, patterns, and lessons learned, and crafting feedback based on the actual text [1]. Chakroun and colleagues developed and piloted quality indicators for narrative feedback that are meant to be operational beyond limited contexts. The seven quality indicators include: 1) providing improvement recommendations, 2) providing a balanced message, 3) comparison against a standard, 4) specificity of feedback, 5) justification for numeric score, 6) use clear language, and 7) non-judgmental style [2]. The EFeCT rubric consists of five elements that were deemed essential to high-quality feedback including: 1) what the learner performed, 2) feedback context, 3) performance assessment, 4) recommendations or needs for improvement, and 5) positive aspects of the performance and improvement suggestions [3].

**Sample Narrative Feedback with Quality Criteria Indicated**

| **LLM-Generated Feedback** | **Criteria** |
| --- | --- |
| Your reflection clearly shows growth in both basic science knowledge and clinical research engagement, and you thoughtfully discuss the tension between empathy and efficiency during patient encounters. You describe concrete actions—redirecting conversations, setting boundaries, and using stress relief strategies—that demonstrate actionable takeaways. Your essay integrates multiple contexts (your academic background, the mentor’s guidance, patient interactions), and you outline specific improvements for year two. To deepen your critical reflection, consider exploring the assumptions that underlie your comfort with empathy (e.g., cultural expectations of physician compassion) and how they shape your boundary setting. Reflect on how patients might interpret your redirects and whether that affects rapport. Finally, think about how your research experience is influencing your professional identity and what concrete commitments you can make to translate these insights into long term practice changes. By interrogating these underlying beliefs and expanding on the implications for your future role, you can move toward a fully critical, transformative reflection. | 1 – Specificity  3 – Reference to Emotions  4 – Lessons Learned  2 – Reflective Ability  7 – Areas for Improvement  5 – Parallel Process  6 – Prompts for Future Consideration |

**References**:

1. Reis SP, Wald HS, Monroe AD, Borkan JM. Begin the BEGAN (The Brown Educational Guide to the Analysis of Narrative)–A framework for enhancing educational impact of faculty feedback to students’ reflective writing. Patient Educ Couns. 2010;80(2):253-259. doi:10.1016/j.pec.2009.11.018
2. Chakroun M, Dion VR, Ouellet K, Graillon A, Désilets V, Xhignesse M, St-Onge C. Quality of narratives in assessment: piloting a list of evidence-based quality indicators. Perspect Med Educ. 2023;12(1):XX. doi:10.1007/s40037-023-00748-4
3. Ross S, Hamza D, Zulla R, Stasiuk S, Nichols D. Development of and preliminary validity evidence for the EFeCT feedback scoring tool. J Grad Med Educ. 2022;14(1):71-79. doi:10.4300/JGME-D-21-00402.1
